# Evaluating an emerging technology-based biorefinery for edible house crickets

**DOI:** 10.3389/fnut.2023.1185612

**Published:** 2023-07-18

**Authors:** Marios Psarianos, Shikha Ojha, Oliver K. Schlüter

**Affiliations:** ^1^Horticultural Engineering, Leibniz Institute for Agricultural Engineering and Bioeconomy (ATB), Potsdam, Germany; ^2^Department of Agricultural and Food Sciences, University of Bologna, Cesena, Italy

**Keywords:** resilient food processing, ultrasound, high pressure, deep eutectic solvent, extraction, fractionation

## Abstract

**Introduction:**

Edible insects, specifically house crickets, are expected to play an important role in the future food systems due to their rich nutritional profile, low environmental impact and growing consumer acceptance as food. Their content of proteins, lipids, chitin and phenolics offer great potential for the valorization of their biomass into nutritional end products and fractions. Furthermore, emerging food processing technologies and green solvents are relevant for improving the valorization process.

**Materials and methods:**

High pressure (HP) and ultrasound (US) processing were implemented in an insect biorefinery system, where a hexane/methanol/water solvent was used to separate fat, phenolics and a solid fraction containing proteins and chitin. Subsequently, a deep eutectic solvent of betaine and urea (B/U) was used to for protein and chitin isolation.

**Results:**

A maximum of 15% of fat was isolated, with no positive effect from the US or HP treatments. The US treatment enhanced the phenolic extraction yield by 38.69%, while HP negatively affected the antioxidant capacity. B/U was efficient in separating proteins and chitin, resulting in a protein concentrate with a protein content ≥80% and a chitinous fraction with a chitin content ≥70%.

**Conclusion:**

House cricket biomass can be refined into valuable fractions with a quick and simple method, making the process industrially relevant.

## 1. Introduction

Edible insects are a promising resource for utilization in the food sector due to their high nutritional value (1) and low environmental impact (2). House crickets (*Acheta domesticus*) are particularly interesting since they have a history of being farmed (3) and are consumed as food and feed in some parts of the world (4). Furthermore, they have been accepted as novel food in the EU (5) and have also been proposed as a food ingredient (6, 7).

A biorefinery refers to the conversion of a biomass feedstock to a number of functional or valuable products (8). Biorefineries are processing facilities that convert biomass into value-added products such as biofuels, biochemicals, bioenergy/biopower, and other biomaterials. Various types of biorefineries have been presented in the literature. Most of them are mainly defined based on individual feedstock, such as corn-based biorefinery, wood-based biorefinery, forest-based biorefinery, palm-based biorefinery, and algae-based biorefinery. However, some researchers and technologists defined biorefineries based on the generation of feedstock, which are first-generation biorefinery (energy crop, edible oil seeds, food crops, and animal fats), second-generation biorefinery (lignocellulosic biomass), and third- or fourth-generation biorefinery (algae and other microbes) (9).

House crickets are characterized by high protein and fat contents (1), phenolic contents (10), and chitin, from which chitosan with antimicrobial properties can be produced (11). These compounds underline the potential of the cricket biomass to be refined into food ingredients, biomaterials, and feed (12). The possibility of insect biorefinery has been explored for some species, including *Tenebrio molitor* (13), *Hermetia illucens* (14), and *Bombyx eri larva* (15). Even though there are no known studies focusing on *Acheta domesticus* biorefinery, the potential of house crickets as a base for the extraction of valuable compounds has been explored (16, 17).

Emerging food processing technologies and green solvents have been suggested to enhance the process of isolation or extraction of valuable compounds from edible insects (18). Ultrasound (US) and pulsed electric fields, for instance, have been shown to increase the fat extraction yield from house crickets (19, 20) and black soldier fly larvae (21). High pressure (HP) can increase the extraction yield of phenolics and proteins from olive pomace (22). Deep eutectic solvents (DESs) offer a successful and environmentally friendly approach for chitin extraction from black soldier flies (23).

House crickets have the potential for conversion into valuable ingredients. A rapid, simple, and waste-reducing biorefinery procedure, which is based on two fractionation steps, was applied to house cricket biomass. Ultrasound and high-pressure treatments, as well as DES, were implemented in the biorefinery to reduce the use of materials that are considered hazardous (24) and to improve the yield of each step. Therefore, the overall aim of this study was to introduce an environmental friendly biorefinery system for the valorization of biomass from house crickets.

## 2. Materials and methods

### 2.1. Sample preparation

Living crickets (*A. domestica*) were purchased from Tropic Shop (Nordhorn, Germany) and were inactivated by freezing at −20^o^C. Afterward, they were thawed at 4^o^C, separated from the frass, washed with water, and oven-dried at 60^o^C until a constant weight was achieved. Dried insects were milled for 10 s using a laboratory mill from Retsch (Haan, Germany) to obtain cricket flour. All chemicals were purchased from Carl Roth GmbH & Co. Kg (Karlsruhe, Germany) unless stated otherwise. Betaine and the standard mixture of amino acids were purchased from Sigma-Aldrich (Merck KGaA, Darmstadt, Germany). Trolox and 2,2-diphenyl-1-picrylhydrazyl were purchased from Alfa Aesar (Massachusetts, United States).

### 2.2. Determination of the sample composition

The moisture content of the flour was determined by the gravimetrical difference of the sample after being placed in a drying oven at 105^o^C for 48 h. The ash content was determined by the gravimetrical difference of the sample after being placed in a furnace at 550^o^C. For the determination of the protein content, 50 mg of the sample was hydrolyzed with 2 ml of HCl of 6 N at 98^o^C for 24 h. The hydrolysates were cooled to room temperature and mixed with 2 ml of NaOH of 6 N and 2 ml of phosphate buffer (720 mM, pH = 6.6). Then, they were diluted to 200 ml and subjected to the analysis of free amino nitrogen content. Briefly, 0.5 ml of the sample, after the appropriate dilution, was mixed with 0.25 ml of ninhydrin color reagent (phosphate buffer 720 mM, pH = 6.6, 0.5% w/v ninhydrin, 0.3% fructose) and incubated at 95^o^C for 20 min. After being cooled down, samples were mixed with 1.25 ml of 0.2% KIO_3_ solution in 40% ethanol, and the absorption was measured at 575 nm with a UV/Vis spectrometer. Bovine serum albumin was used for the calibration curve at a concentration range of 10–40 mg/ml, and the results are expressed as g protein/100 g of sample (25).

Fat content was determined with the Folch method. Briefly, 5 g of insects were homogenized with 100 ml of chloroform/methanol (2:1) solvent at room temperature for 1 h. Afterward, the mixture was centrifuged, and the supernatant was collected and mixed with water at a volume of 0.2 times the volume of the supernatant. The mixture was mixed for 30 more minutes at room temperature and then was centrifuged for 10 min at room temperature and 3,900 × g. The lower phase was collected, and the solvent was removed with a rotary evaporator (Büchi R-100, Flawil, Switzerland). Then, the fat content was determined gravimetrically (26). For the estimation of chitin content, 10 mg of samples were hydrolyzed for 90 min with 0.3 ml of 72% sulfuric acid. Afterward, 8.4 ml of water was added, and the samples were further hydrolyzed for 1 h at 121^o^C. While still warm, 0.5 ml of the samples were taken and mixed with 0.5 ml of NaNO_2_, 1M solution (A), and another 0.5 ml was mixed with 0.5 ml of water (B). These samples were capped and incubated at room temperature for 6 h followed by overnight incubation without a cap. Then, 0.5 ml of 12% ammonium sulfamate was added to both mixtures A and B, followed by thorough vortexing for 4 min. Next, 0.5 ml of 0.5% MBTH solution was added to both A and B mixtures that were then incubated for 1 h at room temperature. Finally, 0.5 ml of FeCl_3_ was added to both A and B mixtures, and after 30 min of incubation, the mixtures were diluted appropriately and their absorbance at 650 nm was measured. Standard chitin was used for the calibration curve at a concentration range of 26–130 μg/ml in the cuvette, and the results were expressed as g chitin/100 g sample (27). For the estimation of total carbohydrates, 90 mg of samples were hydrolyzed for 2 h with 2 ml of 12 M sulfuric acid. Afterward, 10 ml of water was added, and the samples were heated at 98^o^C for 2 h. Then, the samples were cooled down to room temperature, and the hydrolysates were mixed with 6 ml of 10 N KOH and diluted to 100 ml with sodium acetate buffer (200 mM, pH = 5). Total carbohydrate content was estimated on the solutions with the phenol sulfuric acid method (28).

### 2.3. Fractionation process

**Figure 1:**
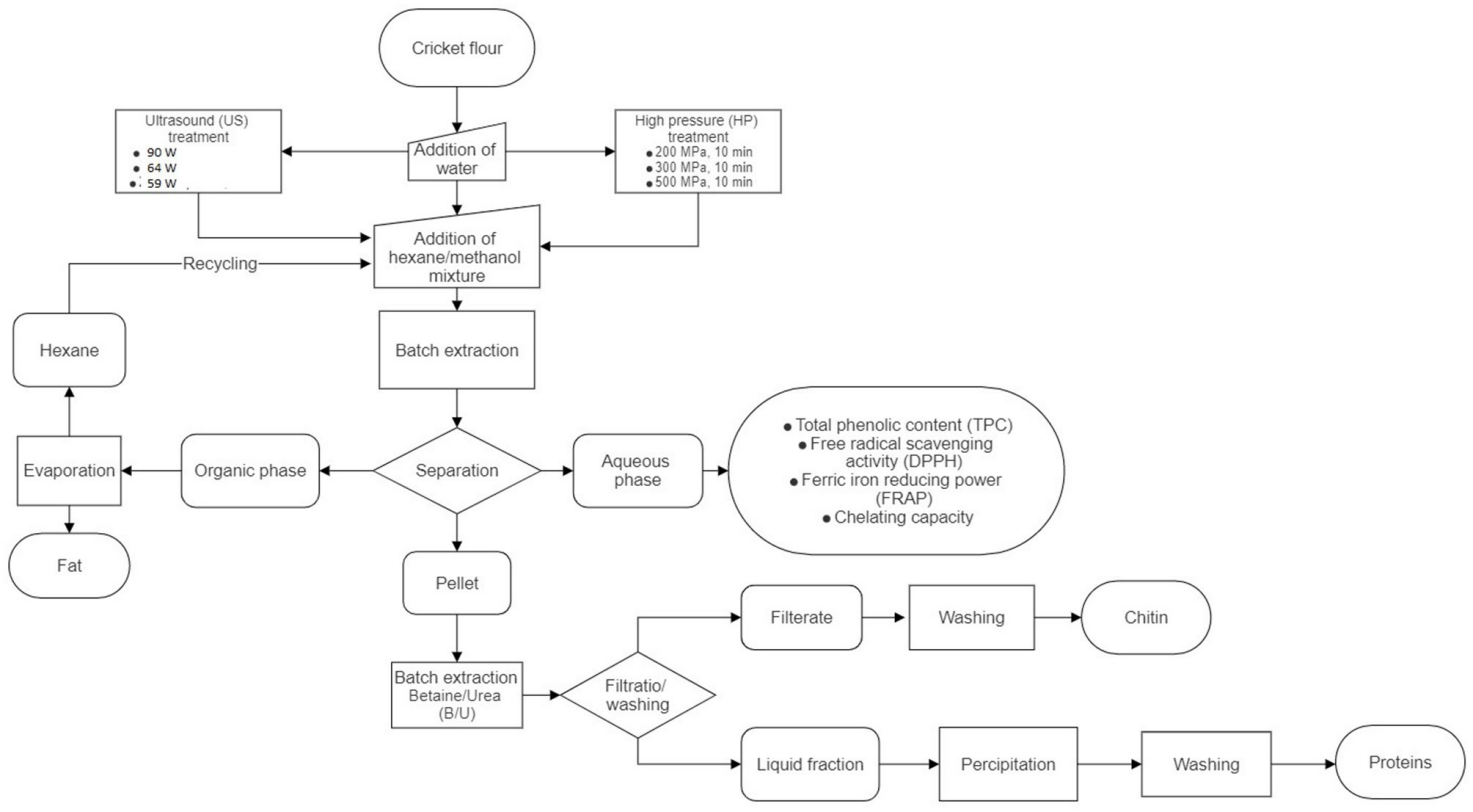
Flow chart of the experimental setup.

The flow chart of the experiments that were performed in the present study is presented in Figure 1. Initially, 5 g of the sample was mixed with 20 ml of distilled water. The US treatment of the samples was performed with a UIP1000hdT unit (Hielscher Ultrasonics GmbH, Teltow, Germany) in accordance with the following conditions: (a) 50% amplitude, 5 min, and 90 W; (b) 25% amplitude, 5 min, and 64 W; and (c) 25% amplitude, 10 min, and 59 W. During the treatments, the samples were placed inside a water bath at 4^o^C to control the temperature increase, which never exceeded 50^o^C for any treatment. The HP treatment of the samples was performed with a mobile high-pressure system U33 (Institute of High Pressure Physics, Warsaw, Poland) that was connected to a water pump, at 200–500 MPa for 10 min, using water as a pressure transmitting medium. A non-treated mixture of sample and water was used as a control.

Afterward, a hexane/methanol (1:1) solvent was added to each sample at different volumes (25 or 50 ml), and the mixture was stirred at room temperature for 1 h (29). The two different volumes of the solvent were chosen with the aim to reduce the amount of required chemicals as much as possible. The selection of the extraction solvent was based on the Folch extraction method, which included the aqueous fraction to remove impurities from the isolated fat (26), but with hexane replacing chloroform, as it is considered less hazardous (24). Afterward, the mixture was centrifuged at 3,900 × g for 10 min, and three phases were generated. The top layer was the organic phase containing hexane and fat, the middle layer was an aqueous-methanolic extract, and the bottom layer was a protein–chitin-rich pellet. After centrifugation, the three phases were separated.

Afterward, the pellet was subjected to a treatment with a DES, composed of betaine and urea (B/U) for 2 h at 80^o^C, as it has been shown to be the most efficient DES for chitin isolation (23). The solvent was prepared by mixing betaine and urea at a molar ratio of 1:2 and agitating the mixture while heating until a clear liquid was formed. After the treatment of the pellet with B/U, the mixture was filtered through a mesh of 0.063 mm, and the filtrate containing chitin was washed with water that was heated to 60^o^C. The filtered liquid that was generated was stored at 4^o^C for 60 min after its pH was modified to 4.5 in order for proteins to precipitate. Afterward, the proteins were isolated via centrifugation at 3,900 × g, 10 min and washed with water with modified pH at 4.5 twice. Both filtrate and protein precipitates were then dried at 60^o^C until constant weight.

### 2.4. Analysis of fractions

#### 2.4.1. Organic phase

The organic phase containing hexane and fat was placed inside a rotary evaporator (Büchi R-100, Flawil, Switzerland) connected to an electric vacuum pump (Büchi V-100, Flawil, Switzerland) operating at 40^o^C and 10 mbar. The hexane was separated from the fat and recycled for further use. The fat extraction yield was determined gravimetrically.

#### 2.4.2. Aqueous phase

##### 2.4.2.1. Total phenolic content

The total phenolic content (TPC) was determined with the Folin–Ciocalteu method. Extracts were diluted appropriately, and then, 0.1 ml of diluted extracts was mixed with 7.9 ml of water. Afterward, 0.5 ml of Folin–Ciocalteu reagent (1 N) was added, and then, 1.5 ml of saturated sodium carbonate solution was added to the mixture. After incubation at 40^o^C for 30 min in the dark, the samples were cooled down to room temperature, and the absorbance was measured with a UV/Vis spectrometer at 765 nm. Methanol was used as a blank control. A gallic acid solution was used for the calibration curve at a concentration range of 100–1,000 mg/L, and the results were expressed as mg gallic acid equivalent (GAE)/100 g of the initial cricket sample (22).

##### 2.4.2.2. Free radical scavenging activity (2,2-diphenyl-1-picrylhydrazyl)

The free radical scavenging activity was determined with the DPPH method. Briefly, 0.1 ml of the appropriately diluted extract was mixed with a freshly prepared 6 × 10^−5^ M DPPH solution and incubated at room temperature in the dark for 15 min. Afterward, the absorbance was measured at 515 nm, using methanol as a blank control. The antioxidant activity is correlated with the difference in absorbance between the blank control and the sample. Trolox was used for the calibration curve at a concentration range of 0.1–1 mM, and the results are expressed as mM Trolox equivalent (mM TE) (30).

##### 2.4.2.3. Ferric iron reducing power

Ferric iron reducing power (FRAP) was estimated by mixing 0.5 ml of an appropriately diluted extract with 0.5 ml of sodium phosphate buffer (0.2 M, pH = 6.6) and 0.5 ml of 1% potassium ferricyanide. The mixture was incubated at 50^o^C for 20 min, and then, 0.5 ml of 10% trichloroacetic acid was added. Afterward, 2 ml of water and 0.4 ml of ferric chloride 0.1% were added. Samples were vortexed, and the absorbance was measured at 700 nm. A higher absorbance indicates a stronger antioxidant activity. Methanol was used as a blank control, and Trolox was used for the calibration curve at a concentration range of 50–500 μM. The results are expressed as mM TE (31).

##### 2.4.2.4. Chelating capacity

Chelating capacity was estimated by mixing 0.1 ml of an appropriately diluted extract with 3.7 ml of methanol and 0.1 ml of 2 mM FeCl_2_. After incubating the samples for 3 min at room temperature, 0.2 ml of 5 mM ferrozine was added. The samples were then incubated for another 10 min at room temperature, and the absorbance was measured at 562 nm. Methanol was used as a blank control. The antioxidant activity is correlated with the difference in absorbance between the blank and the sample. EDTA was used for the calibration curve at a concentration range of 0.25–2 mg/ml, and the results are expressed as mg EDTA equivalent/ml (32).

#### 2.4.3. Filtrate

Regarding the filtrate, the protein and chitin contents were determined with the methods described in Section 2.2. The filtrates were further analyzed directly with FTIR using a Nicolet iS5 spectrometer (Thermo Scientific, US-WI 53711 Madison, USA).

#### 2.4.4. Protein precipitate

Similarly, the protein and chitin contents of the protein precipitate were analyzed with the methods that are described in Section 2.2. Additionally, the amino acid (AA) profile was estimated as follows: 0.1 g of each sample was hydrolyzed for 24 h at 110^o^C using an aqueous hydrolysis solution that was prepared as follows: 0.5 g of phenol was mixed with 200 ml of water and then 66 mg of norleucine and 250 ml of 6 N HCl were added. After the hydrolysis, 0.2 ml of the hydrolysate was removed, dried, and mixed with 1 ml of lithium dilution buffer (650-0018, MembraPure GmbH, Hennigsdorf Germany). Then, the samples were injected in an Aracus Classic amino acid analyzer (MembraPure GmbH, Hennigsdorf Germany) with an ion exchange chromatographic column (125 × 3 mm), an autosampler with 100 μl loop, and a reactor for post-column derivatization with ninhydrin. The samples were detected at 570 nm and 440 nm for proline. The duration of the analysis was 90 min (33).

### 2.5. Statistical analysis

Each fractionation process was repeated in triplicate, and each analytical method used for each fraction was repeated in triplicate as well. Error bars on the graphs indicate standard errors. Significant differences among the data collected from the samples that were processed with different methods were identified with a one-way analysis of variance (ANOVA), and Tukey's test was applied *post hoc* for mean separation at a confidence level of 0.05. Normality was tested with the Shapiro–Wilk test, and data that did not follow normal distribution were normalized prior to the analysis. All analyses were performed using IBM SPSS Statistics 23 (IBM Corp., Armonk, N.Y., USA).

## 3. Results

### 3.1. Composition of the material

The estimated composition of the biomass of house crickets (Table 1) confirms their potential for the utilization of their ingredients in the food sector. As has been suggested by Rumpold and Schlüter (1), Psarianos et al. (19), and Udomsil et al. (34), crickets were found to have high protein and lipid contents, as well as a significant amount of chitin. The variation of the values of the insect composition among different studies can be attributed to different rearing procedures and feeding substrates of the targeted insects (35).

**Table 1:** Composition (g/100 g dry weight) of the adult house cricket flour. Data are expressed as mean ± SD (*n* = 3).

**Compounds**	**g/100 g dry weight**
Proteins	55.58 ± 3.29
Crude fat	17.45 ± 0.62
Ash	4.17 ± 0.34
Carbohydrates	4.52 ± 0.15
Chitin	8.80 ± 1.26

### 3.2. Fat isolation

**Figure 2:**
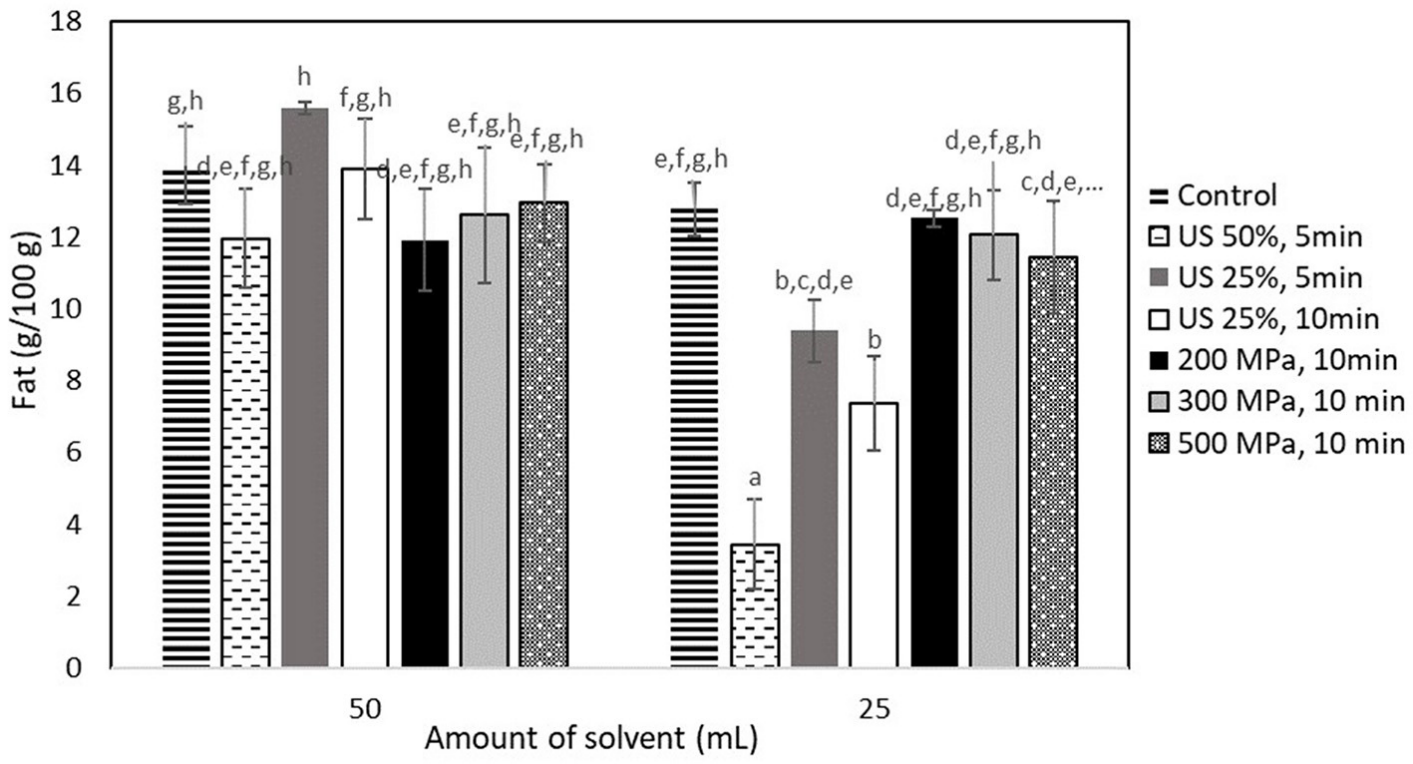
Fat extraction yield (g/100 g sample) obtained from samples treated by US and HP at different conditions. Error bars indicate standard errors among replicates of the same process. Superscript letters (a, b, …) indicate significant differences between the means obtained from different samples.

As shown in Figure 2, neither US nor HP processing had a positive effect on the fat extraction yield. The highest yield obtained from the US treated samples at 90 W (50% amplitude, 5 min) mixed with 50 ml of the extraction medium was 15.61 ± 0.17 g fat/100 g cricket flour, which was still not significantly higher (*p* < 0.05) than the yield obtained from the control sample (14.00 ± 1.07 g fat/100 g cricket flour). A similar trend was also observed for the process pathways that required 25 ml of the extraction medium, with the highest yield being observed for the control sample (12.78 ± 0.74 g fat/100 g cricket flour). The fat yield obtained from the control sample that was mixed with the 50 ml of the extraction medium was 80.23% of the total fat (Table 1). This means that the fat extraction process was quite efficient without any pretreatment with HP or US.

Regarding the untreated samples that were subjected to extraction at different solvent volumes of 25 and 50 ml, no significant difference was observed (*p* < 0.05), with a yield of ~13 g fat/100 g of cricket flour. However, the US-treated samples that were mixed with 25 ml of the extraction solvent showed a significantly negative yield (*p* < 0.05), with the lower yield being obtained from the one treated with 50% amplitude for 5 min (3.44 ± 1.25 g fat/100 g insect flour).

### 3.3. Aqueous extract

**Figure 3:**
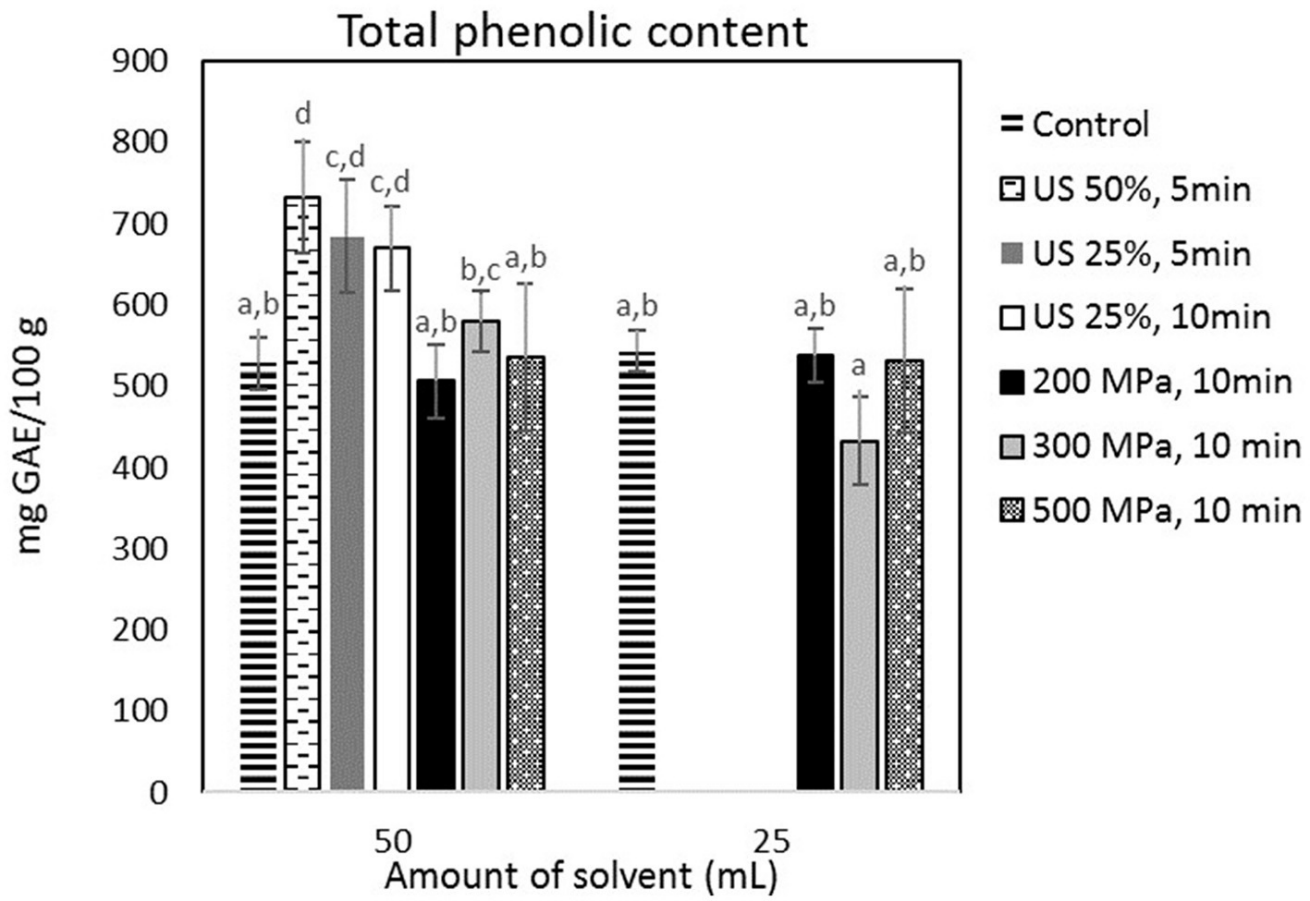
TPC (mg GAE/100 g sample) obtained from the samples treated by US and HP at different conditions. Error bars indicate standard errors among replicates (*n* = 9) of the same measurement. Superscript letters (a, b, …) indicate significant differences between means obtained from different samples.

Figure 3 shows that HP processing had no effect on the extraction yield of phenolic compounds (*p* < 0.05) with the control extraction process using 50 and 25 ml of extraction medium leading to a 528.08 ± 32.49 mg and 543.09 ± 25.75 mg GAE/100 g of cricket flour, respectively. US treatment at all tested conditions did nevertheless lead to a significant (*p* < 0.05) increase of the TPC in the aqueous extract. The highest yield of phenolic compounds was obtained, after treatment with US at 90 W and was equal to 732.38 ± 68.94 mg GAE/100 g cricket flour. In that case, US treatment increased the TPC in the extract by 38.69%, compared to the untreated sample that was mixed with the same amount of solvent. However, the US-treated sample showed the highest TPC compared to every sample.

**Figure 4:**
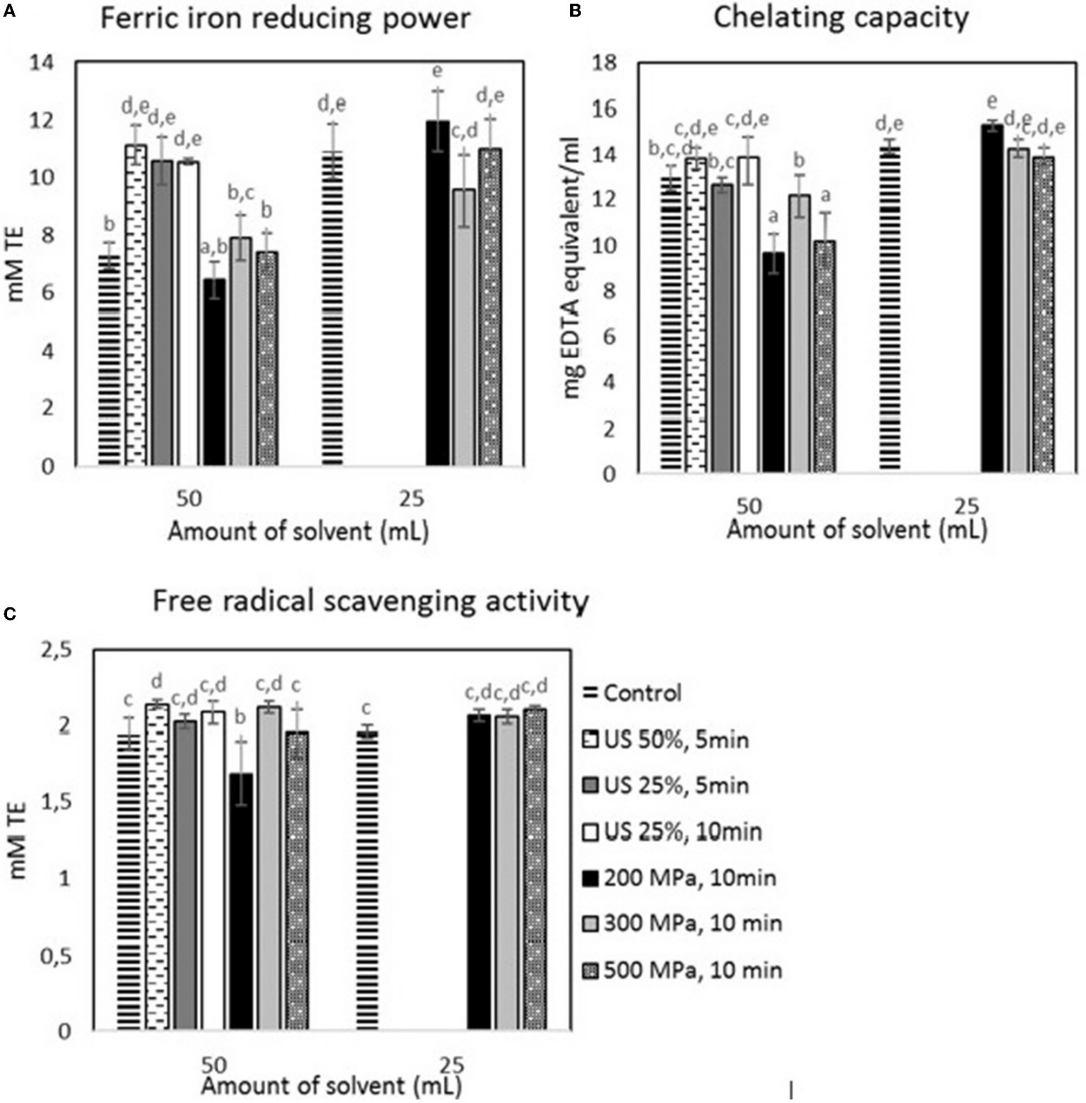
Antioxidant activity estimated for the samples treated by US and HP at different conditions: **(A)** FRAP, **(B)** chelating capacity, and **(C)** DPPH. Error bars indicate standard errors among replicates (*n* = 9) of the same measurement. Superscript letters (a, b, …) indicate significant differences between means obtained from different samples.

According to Figure 4, a similar trend to the TPC was observed for the FRAP and free radical scavenging activity, where US treatment did increase the antioxidant activity of the aqueous extracts. In specific, the samples mixed with the higher solvent volume showed a 52.40% (*p* < 0.05) and a 9.81% (*p* < 0.05) increase in their FRAP and free radical scavenging activity, respectively, after the US treatment with 50% amplitude for 5 min. However, the samples subjected to the higher solvent volume showed no significant differences after HP processing, apart from the treatment with 200 MPa for 10 min that reduced the radical scavenging activity by 15.38% (*p* < 0.05). Furthermore, there was no positive effect of US and HP on the chelating capacity. Nevertheless, HP processing significantly (*p* < 0.05) reduced the chelating capacity of the samples treated with 200 and 500 MPa and subjected them to the higher solvent volume. Furthermore, decreasing the solvent volume by 50% without any pretreatment resulted in a significant (*p* < 0.05) increase in the FRAP of the extracts but did not affect the radical scavenging (*p* > 0.05) and chelating activity (*p* > 0.05). Finally, the radical scavenging activity of the US-treated sample at 50% amplitude for 5 min was the highest among all samples.

### 3.4. Chitin isolation

**Figure 5:**
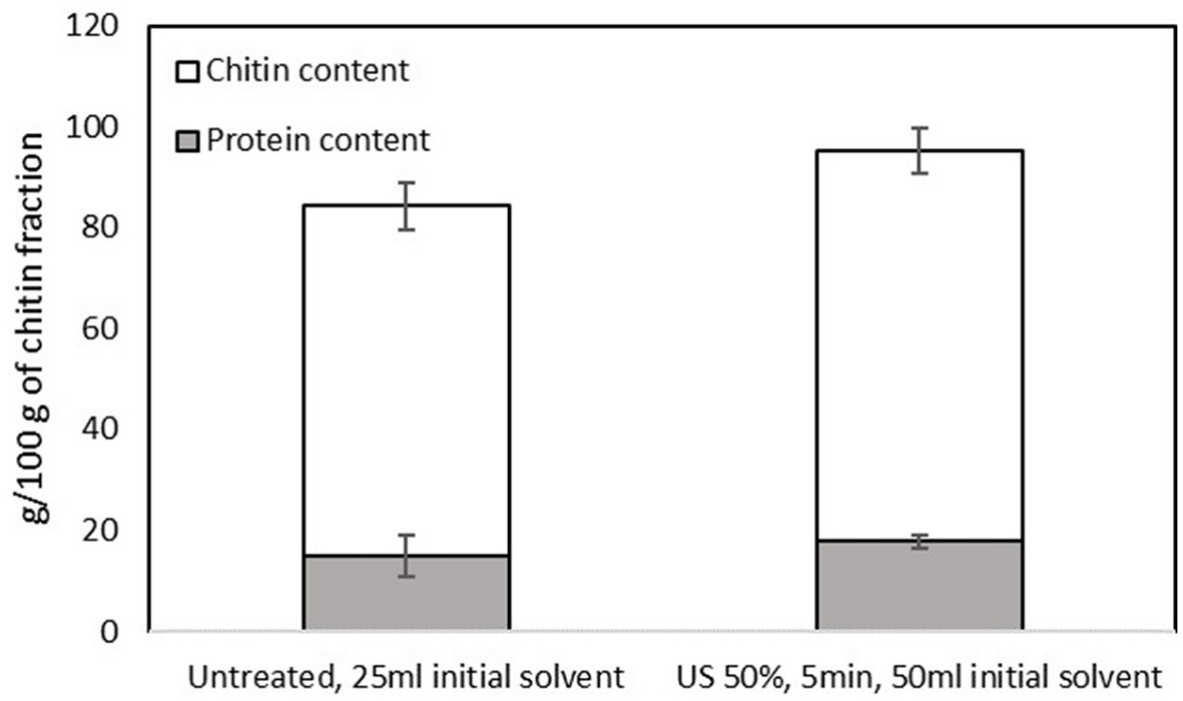
Protein and chitin contents of the chitin-rich fraction obtained from the pellet of the untreated sample that was mixed with 25 ml of the initial solvent and the US-treated sample with a 50% of amplitude for 5 min that was mixed with 50 ml of the initial solvent. Error bars indicate standard errors among replicates (*n* = 9) of the same measurement. Significant differences (*p* > 0.05) among means are indicated with the uppercase and lowercase of the same superscript letters (a and A, b and B).

**Figure 6:**
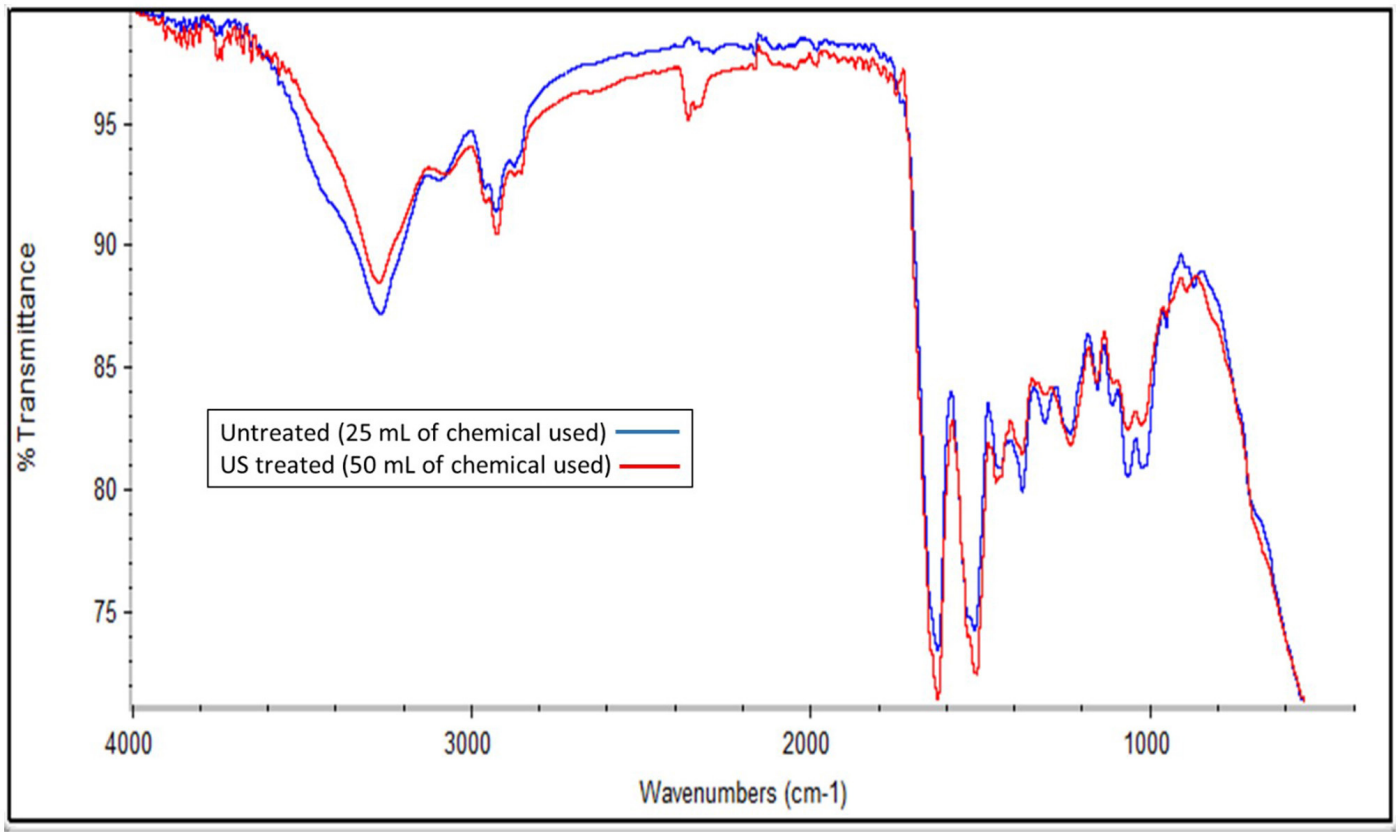
FTIR spectra of the chitin-rich fraction obtained from the pellet of the untreated sample that was mixed with 25 ml of the initial solvent and the US-treated sample with a 50% of amplitude for 5 min that was mixed with the 50 ml of the initial solvent.

The treatment with B/U was successful in isolating a chitin-rich fraction from both samples (Figure 5). The chitin fractions have a high chitin content (~70 g/100 g of filtrate) and a low content of protein impurities (< 20 g/100 g filtrate). This is considered sufficient since an incomplete deproteinization and thus the existence of protein impurities in chitin does not affect the properties of chitosan (36). The application of NADES for chitin extraction from insects has previously been reported to lead to a chitinous fraction with a purity of 70%−90%, depending on the solvent (23). The chitin content in the filtrate obtained from the US-treated sample was found to be significantly higher (*p* < 0.05) than that of the untreated one (77.44 ± 4.41 and 69.45 ± 4.68 g chitin/100 g filtrate, respectively). The FTIR spectrum of the two materials (Figure 6) is almost identical and has all the characteristic peaks that have been observed for insect chitin (Table 2) (11, 37).

**Table 2:** Characteristic peaks identified for the chitin-rich fractions obtained from the pellet of the untreated sample that was mixed with 25 ml of the initial solvent and the US-treated sample with a 50% of amplitude for 5 min that was mixed with the 50 ml of the initial solvent.

**Wavelength (cm^−1^)**	**Bond**
1,025	C–O asym. stretching in the phase ring
1,066	C–O–C asym. stretch in the phase ring
1,157	Asymmetric bridge oxygen stretching
1,309	Waging of CH_2_ of amide III
1,376	Symmetrical deformation mode of CH_3_
1,446	Bending of CH_2_
1,509	N–H bend and N–C stretch
1,625	C=O stretch of amide I
2,925	C–H stretching of CH_2_ and CH_2_OH groups
3,270	N–H stretching

### 3.5. Protein isolation

**Figure 7:**
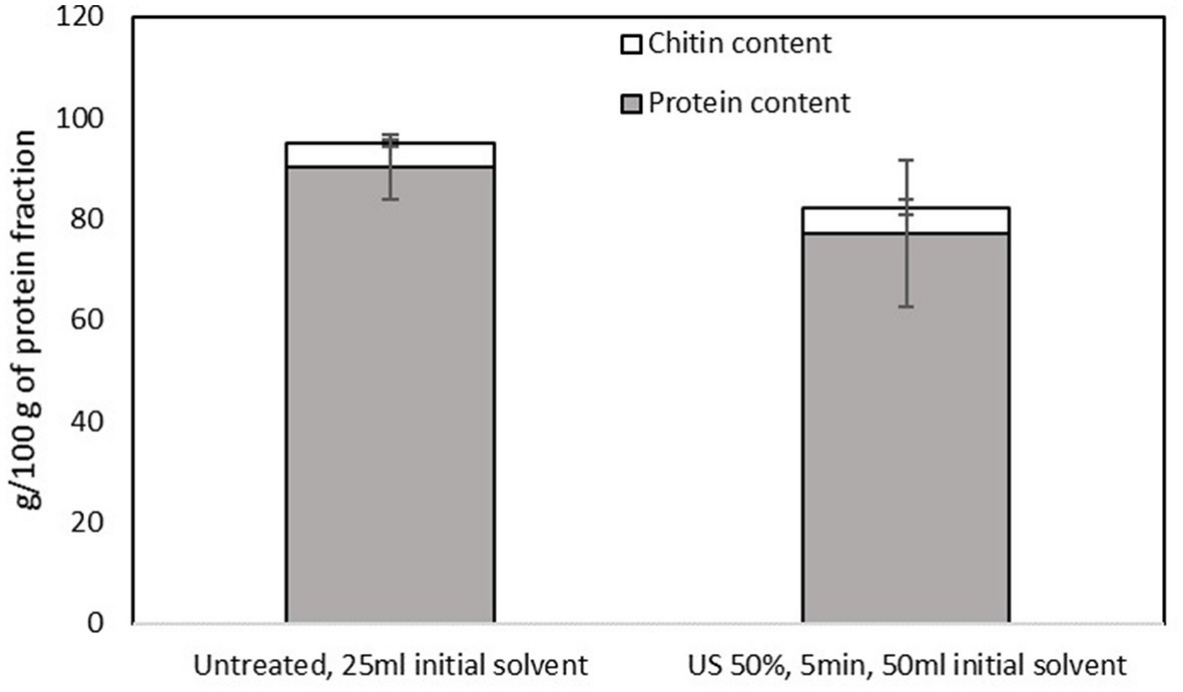
Protein and chitin contents of the protein-rich fraction obtained from the pellet of the untreated sample that was mixed with 25 ml of the initial solvent and the US-treated sample with a 50% of amplitude for 5 min that was mixed with 50 ml of the initial solvent. Error bars indicate standard errors among replicates (*n* = 9) of the same measurement. Significant differences (*p* > 0.05) among means are indicated with the uppercase and lowercase of the same superscript letters (a and A, b and B).

The efficiency of the separation of proteins and chitin in the pellet was also evident in the protein fraction. The protein content of the precipitate was high (ranging from 77 to 90 g/100 g of precipitate) for both samples, without significant differences among them (*p* > 0.05), as shown in Figure 7. Regarding the AA profile of the samples that are presented in Table 3, both samples contain both essential and non-essential amino acids, without any significant differences among samples, while ammonia was observed (~1,805.55 ± 58.85 μmol/l), which was considered a by-product of the treatment with urea. The amino acids that are commonly reported in fractions from house crickets (1, 38) were also estimated in the present study. Additionally, it was observed that the protein precipitates contained norleucine, beta-alanine, g-aminobutyric acid, 1-methyl-histidine, carnosine, and ornithine.

**Table 3:** Amino acids (μmol/l) that were identified in the hydrolysate from the protein-rich fraction obtained from the pellet of the untreated sample that was mixed with 25 ml of the initial solvent and the US-treated sample with a 50% of amplitude for 5 min that was mixed with 50 ml of the initial solvent.

**Amino acids (μmol/l)**	**Untreated (25 ml of chemical used)**	**US treated (50 ml of chemical used)**	***p-*value**
Aspartic acid	1,493.81 ± 190.60	1,302.79 ± 95.97	0.196
Threonine	1,032.96 ± 77.01	950.94 ± 76.77	0.261
Serine	1,447.58 ± 185.25	1,275.40 ± 105.99	0.235
Glutamic acid	2,436.31 ± 246.96	2,206.47 ± 77.69	0.199
Proline	2,530.67 ± 155.91	2,355.77 ± 261.48	0.376
Glycine	1,879.11 ± 3.93	1,783.03 ± 122.65	0.247
Alanine	3,012.21 ± 136.98	2,905.84 ± 338.83	0.641
Valine	1,568.94 ± 42.85	1,496.00 ± 162.74	0.484
Cysteine	119.83 ± 25.37	95.57 ± 23.92	0.295
Methionine	316.71 ± 46.63	268.33 ± 12.17	0.119
Isoleucine	1,037.48 ± 79.67	937.20 ± 58.93	0.155
Leucine	1,853.38 ± 125.49	1,677.59 ± 98.22	0.129
Norleucine	203.13 ± 6.57	205.91 ± 6.83	0.637
Tyrosine	895.64 ± 17.56	839.09 ± 68.16	0.236
Phenylalanine	627.99 ± 77.52	552.55 ± 41.85	0.212
Beta-alanine	2,605.11 ± 650.79	4,263.19 ± 804.94	0.171
G-aminobutyric acid	160.90 ± 21.44	163.98 ± 25.43	0.880
Histidine	414.86 ± 19.85	385.08 ± 23.76	0.171
1-Methyl-histidine	15.74 ± 1.08	15.62 ± 4.24	0.965
Tryptophan	22.92 ± 0.44	22.68 ± 4.90	0.938
Carnosine	49.19 ± 5.90	45.45 ± 15.92	0.722
Asparagine	565.04 ± 53.14	292.17 ± 174.72	0.061
Ornithine	7.26 ± 0.42	6.75 ± 4.70	0.860
Lysine	745.89 ± 0.00	745.83 ± 251.81	0.090
Arginine	1,074.67 ± 88.42	965.29 ± 52.08	0.130

Data are presented as mean ± SD (n = 9).

## 4. Discussion

### 4.1. Fat fraction

The refining process that the present study suggests to fractionate the cricket flour is already quite effective when targeting the fat yield, so US processing could not cause a further increase, as it was reported for other fat extraction methods (20). HP treatment has been reported to negatively affect fat extraction when treated at intense HP conditions, which was attributed to the disruption of triglyceride structure (39). Therefore, a lack of enhancement of the fat extraction due to HP treatment could be expected. However, the potential of HP for the facilitation of the extraction of phenolics was further investigated.

Regarding the samples mixed with 25 ml of the solvent, the negative effect of US could be attributed to the generation of an emulsion that was observed after the US treatment in all cases and that could not be broken by the addition of the chemical solvent at a lower volume. US treatment has been reported to be used for the generation of emulsions and to improve emulsifying properties (40). This is mainly attributed to the breaking of oil and emulsion droplets, generating a fine emulsion (41) or the formation of a micro-jet during cavitation that would push water droplets in the oil phase (42).

Based on these results, it was considered that the fractions of the US-treated samples that were mixed with the 25 ml of the solvent should not be further evaluated, since the treatment had a negative effect on the generation of the fat fraction.

### 4.2. Aqueous extract

US treatment has previously been reported to enhance the extraction of phenolic compounds from insects (43) and other materials (44, 45), which was also confirmed in the present study. Furthermore, HP treatment has been reported to enhance phenolic extraction in plant oils (46) and TPC in oils extracted from insects (39). However, in the present study, HP did not yield any increase in phenolics in the extract. The different results that were observed by (39) may be attributed to the fact that the increase in the phenolic yield, which they observed is estimated on the isolated oil and not in an insect-based extract oil. Furthermore, in the present study, the tested HP treatment conditions were milder in terms of operating pressure and treatment time compared to the treatments used by Ugur et al. (39). This could also attribute to no increase of TPC in the present study, since it has been shown that the yield of TPC can be affected by both pressure and treatment time (22).

The enhancement of the antioxidant activity of extracts that are obtained from food materials with the implementation of US treatment was expected due to the increased TPC, since the antioxidant activity can be linked to the TPC (22).

Based on the results that are presented in Section 3.3, HP was considered inappropriate for implementation in the insect biorefinery. HP treatment has been reported to disrupt the structures of triglycerides in house crickets and mealworms, thus having a negative effect on fat extraction (39). In the present study, no negative effect of HP on the extraction yield of the cricket compounds was observed, which could be attributed to the lower pressure and shorter duration of the HP treatment. However, considering that HP did not improve the extraction yield of both lipids and phenolics from the house crickets, the possibility of implementing HP processing in the process of isolation of valuable compounds from crickets was rejected. On the contrary, the application of US treatment showed potential. Regarding the samples subjected to 50 ml of the initial extraction solvent, the one treated with US at 90 W was considered the most promising. Regarding the ones subjected to the 25 ml of the initial solvent, the untreated one was considered the most promising. Therefore, the pellet from these two samples was collected and subjected to the treatment with B/U to obtain chitin and a protein concentrate.

### 4.3. Chitin fraction

DESs have been successfully implemented in the process of chitin isolation from different materials, including crustacean waste (47) and insects (23). In particular, B/U has been tested for chitin extraction, among a variety of DES, from *H. illucens* and was reported to be the most efficient, with high degrees of demineralization and deproteinization (23). The significantly different chitin contents of the two filtrates (*p* > 0.013) could partially explain the slight differences in the FTIR peak intensities (Figure 6), while the improvement of chitin extraction via US processing has also been observed for squid pens (48).

### 4.4. Protein fraction

The importance of some of the observed amino acids is related to their properties. Beta-alanine has been reported to positively affect body performance (49), same as ornithine (50), and g-aminobutyric acid has been reported to have various benefits on sleep, stress, and blood pressure reduction (51), while carnosine has been suggested as a functional food ingredient for its antioxidant properties (52).

The ability of B/U to cause the isolation of chitin and the removal of other fractions is related to the structure of the DES (23). The DESs are formed from salts that work as hydrogen bond donors and acceptors forming hydrogen bonds, with a high melting point, which when mixed together at a particular molar ratio lead to a depression of the melting point (53, 54). During the treatment of the sample with the B/U, the hydrogen bond donor and receiver molecules dissolve chitin by disturbing its structure and forming new bonds (55). During filtration and washing, the insoluble chitin remains in the filtrate, while the proteins are mostly in the liquid and can be isolated with precipitation.

## 5. Conclusion

House cricket flour was found to be an appropriate substrate for insect biorefinery with a simple and quick process. All main fractions (lipids, proteins, and chitin) were recovered successfully, while an antioxidant and a phenolic-rich extract were also generated. Both HP and US were found to be inefficient to increase the fat extraction yield; however, US treatment increased the yield of phenolics and enhanced the antioxidant activity of the aqueous extract. Furthermore, chitin was successfully separated from proteins using DES. No effect of HP on the fat extraction could be attributed to the triglyceride structure (39), while the negative effect of US on the fat extraction of some of the samples was attributed to an observed emulsion that was the result of the US process. However, further studies are needed to explore the potential of these pretreatments on the biomass of house crickets.

Using a lower volume of the initial solvent (25 ml instead of 50 ml) without any pretreatment showed a comparable extraction efficiency when combining a higher volume (50 ml) with US treatment at 90 W. Furthermore, the process pathway implementing the US treatment offered the advantage of higher antioxidant ability of the aqueous fraction. Due to the short duration of each sequential process and the successful isolation of all main fractions from the house crickets, the processing pathway implemented in the present study presents the potential to be applied to an industrial scale.

## Data availability statement

The raw data supporting the conclusions of this article will be made available by the authors, without undue reservation.

## Author contributions

MP: research design, data collection and analysis, and writing and review—editing. SO: research design and writing and review—editing. OS: writing and review—editing and project administration. All authors contributed to the article and approved the submitted version.
